# Supplementary material for: Phylodynamic analysis reveals disparate transmission dynamics of Mycobacterium tuberculosis complex lineages in Botswana
Source: Sci Rep. 2025 Dec 23;16:1813. doi: 10.1038/s41598-025-31425-z (PMC12804184; doi:10.1038/s41598-025-31425-z)
Supplement: Supplementary file 1 — Supplementary Material 1 [file 41598_2025_31425_MOESM1_ESM.docx]

**Phylodynamic analysis reveals disparate transmission dynamics of *Mycobacterium tuberculosis* complex lineages in Botswana: Supplementary Appendix**

**S1 Fig.** Inferred effective population size over time for individual *Mycobacterium tuberculosis* complex lineage in Botswana.

**S2 Fig.** Maximum clade credibility tree of *Mycobacterium tuberculosis* complex lineage 1 with 95% highest posterior density intervals of node heights.

**S3 Fig.** Maximum clade credibility tree of *Mycobacterium tuberculosis* complex lineage 2 with 95% highest posterior density intervals of node heights.

**S4 Fig.** Maximum clade credibility tree of *Mycobacterium tuberculosis* complex lineage 4.1.1 with 95% highest posterior density intervals of node heights.

**S5 Fig.** Maximum clade credibility tree of *Mycobacterium tuberculosis* complex lineage 4.1.2 with 95% highest posterior density intervals of node heights.

**S6 Fig.** Maximum clade credibility tree of *Mycobacterium tuberculosis* complex lineage 4.3.2 with 95% highest posterior density intervals of node heights.

**S7 Fig.** Maximum clade credibility tree of *Mycobacterium tuberculosis* complex lineage 4.3.4 with 95% highest posterior density intervals of node heights.

**S8 Fig.** Maximum clade credibility tree of *Mycobacterium tuberculosis* complex lineage 4.4 with 95% highest posterior density intervals of node heights.

**S9 Fig.** Maximum clade credibility tree of *Mycobacterium tuberculosis* complex lineage 4.8 with 95% highest posterior density intervals of node heights.

**S10 Fig.** Maximum likelihood phylogeny of Mycobacterium tuberculosis complex (Mtbc) isolates by sampled location in Botswana, 2012–2016 (n = 1,354).

**S11 Fig.** Cluster size distribution (based on a 5-SNP cutoff) by Mycobacterium tuberculosis complex (Mtbc) lineages and sampling location in Botswana, 2012-2016

**S1 Table.** Posterior clock rate of the *Mycobacterium tuberculosis* complex lineages.

**S2 Table.** Genomic cluster proportions (based on a 12-SNPs cutoff) of the *Mycobacterium tuberculosis* complex lineages.

**S3 Table.** HIV prevalence by *Mycobacterium tuberculosis* complex lineages in Botswana, 2012–2016.

**S4 Table.** Unique single nucleotide polymorphisms (SNPs) distinguishing *Mycobacterium tuberculosis* complex lineages L4.3.2 and L4.3.4 in Botswana.

S1 Fig. Inferred effective population size over time for individual *Mycobacterium tuberculosis* complex lineage in Botswana
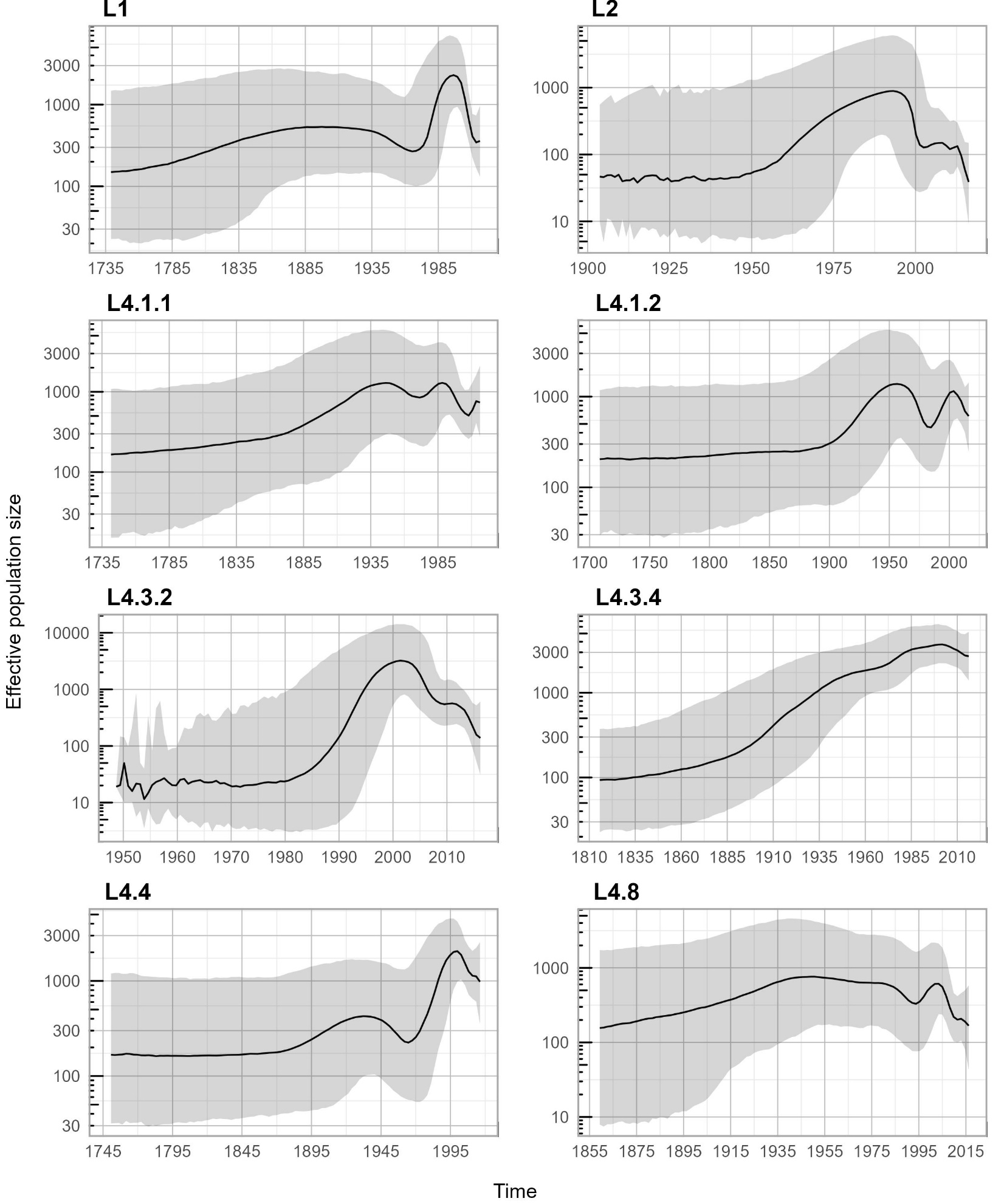


S2 Fig. Maximum clade credibility tree of *Mycobacterium tuberculosis* complex lineage 1 with 95% highest posterior density intervals of node heights. Tree tips are colored by the location of the sampled isolates.


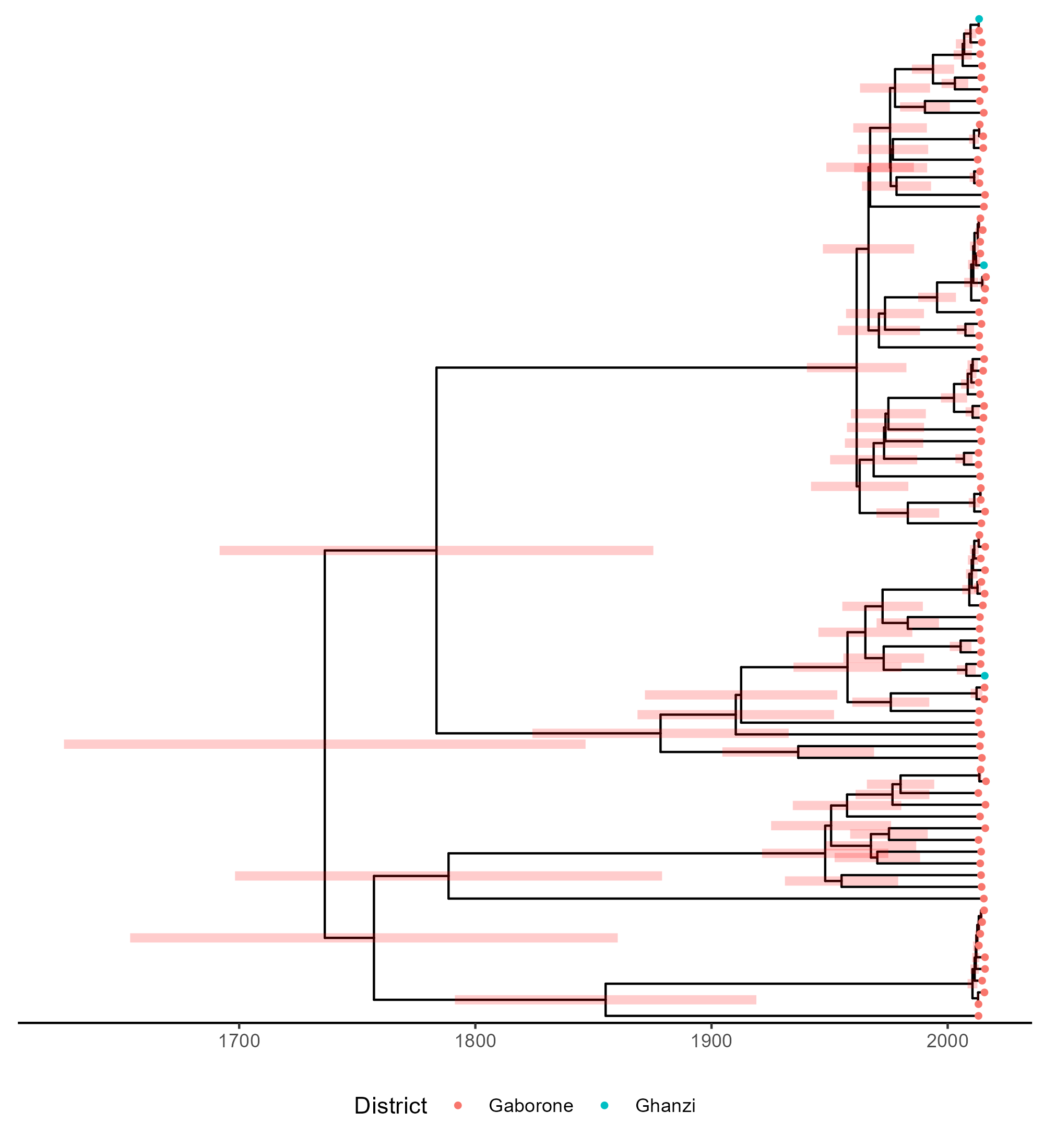


S3 Fig. Maximum clade credibility tree of *Mycobacterium tuberculosis* complex lineage 2 with 95% highest posterior density intervals of node heights. Tree tips are colored by the location of the sampled isolates.


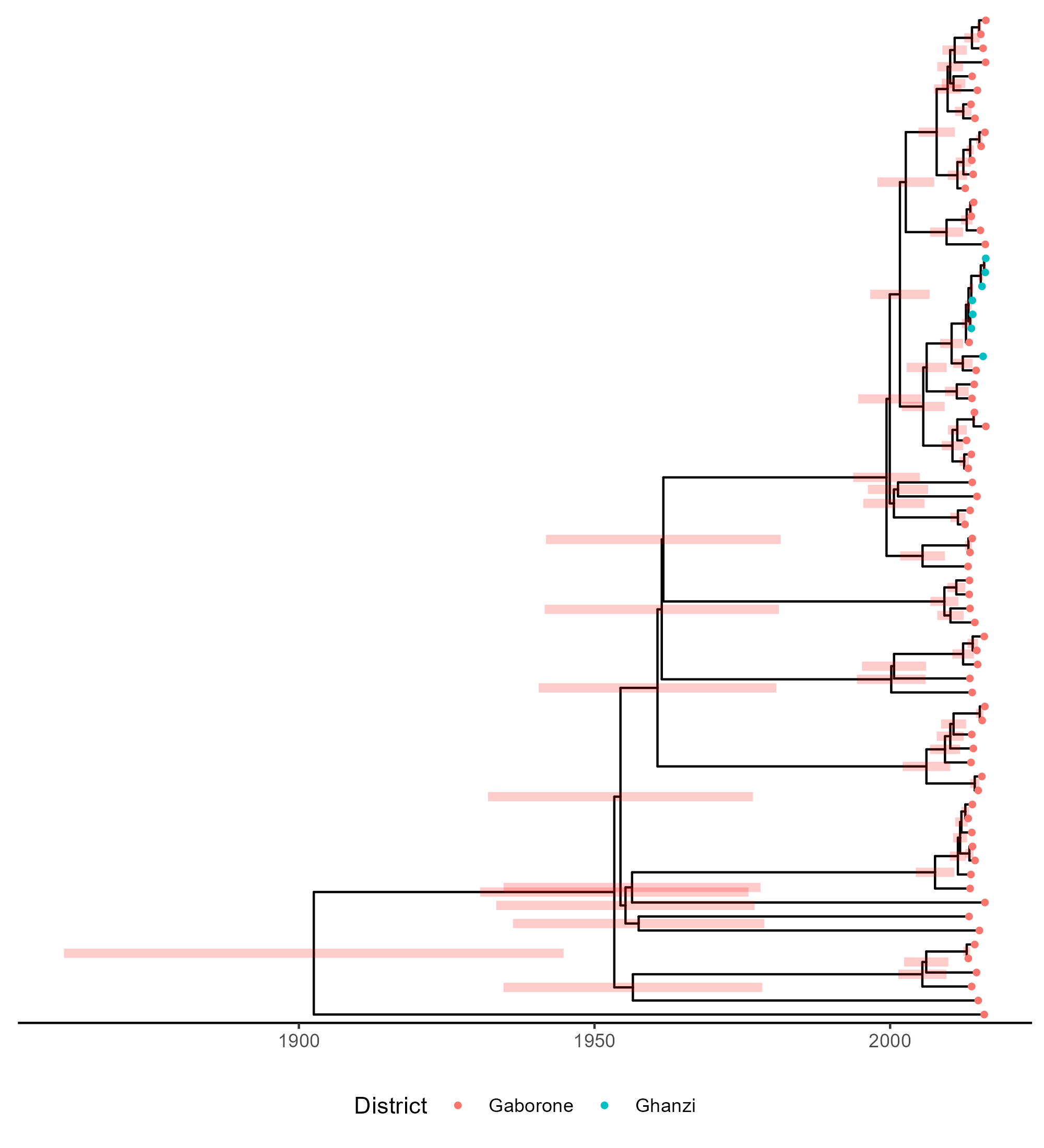


S4 Fig. Maximum clade credibility tree of *Mycobacterium tuberculosis* complex lineage 4.1.1 with 95% highest posterior density intervals of node heights. Tree tips are colored by the location of the sampled isolates.


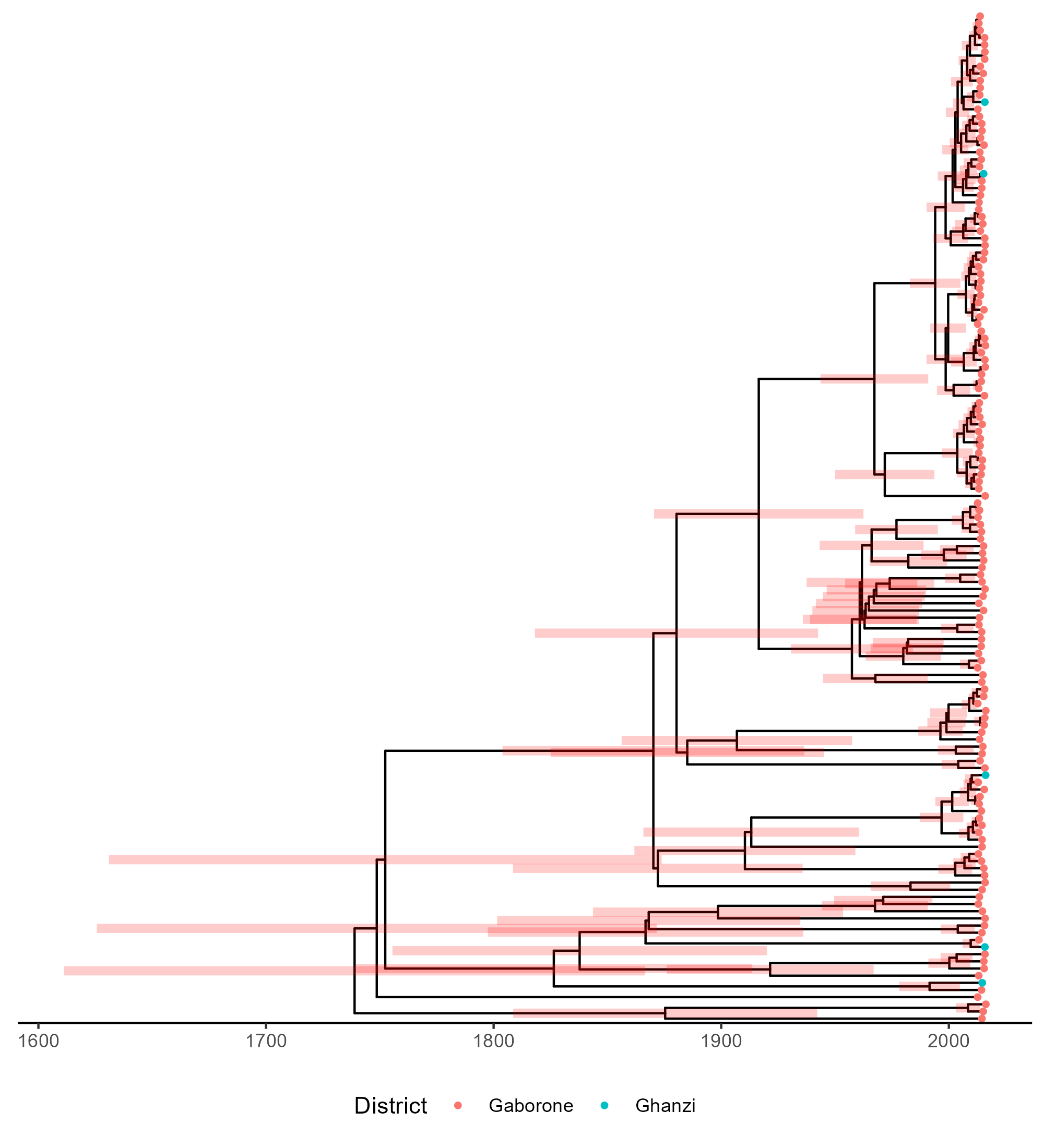


S5 Fig. Maximum clade credibility tree of *Mycobacterium tuberculosis* complex lineage 4.1.2 with 95% highest posterior density intervals of node heights. Tree tips are colored by the location of the sampled isolates.


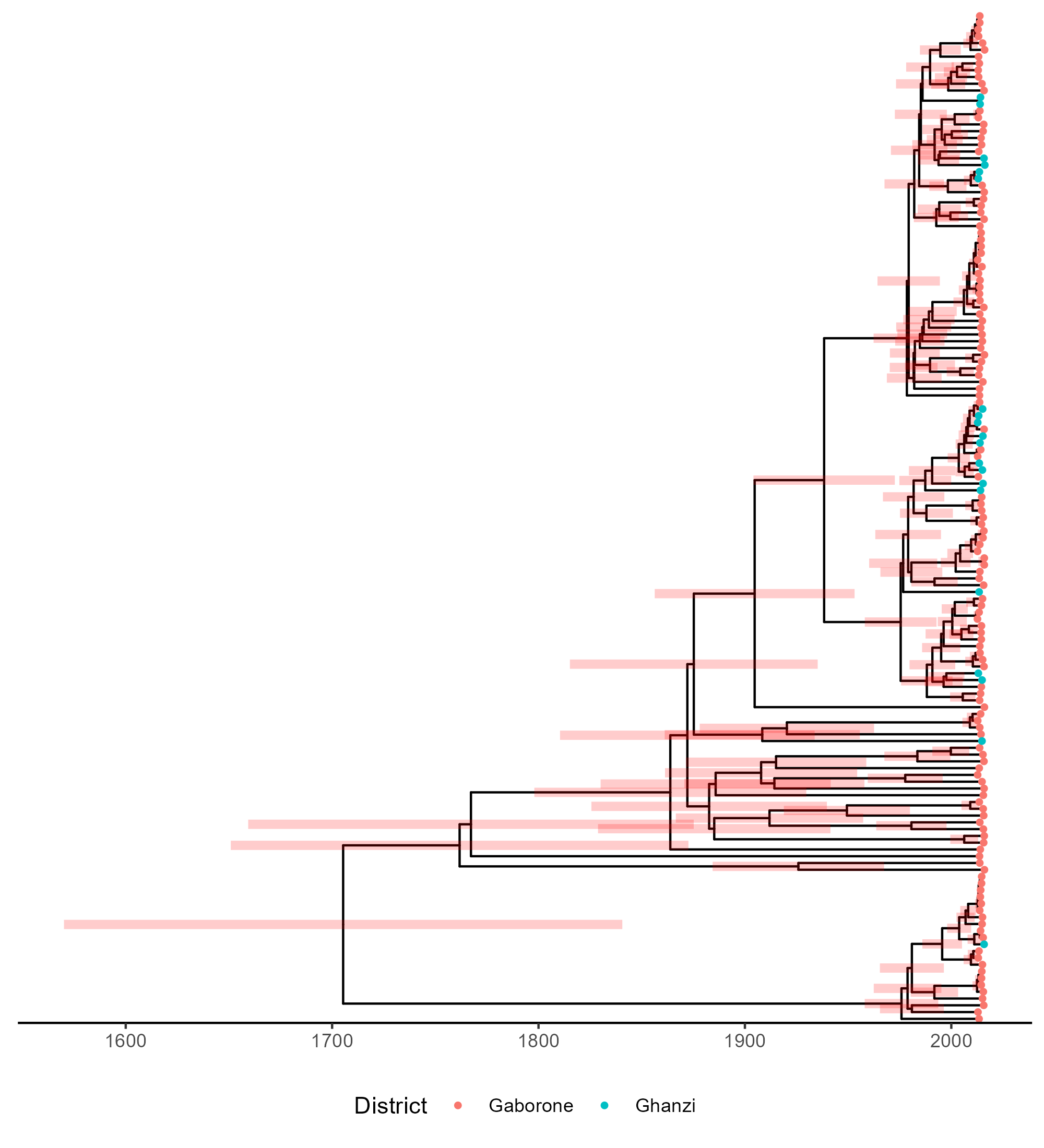


S6 Fig. Maximum clade credibility tree of *Mycobacterium tuberculosis* complex lineage 4.3.2 with 95% highest posterior density intervals of node heights. Tree tips are colored by the location of the sampled isolates.


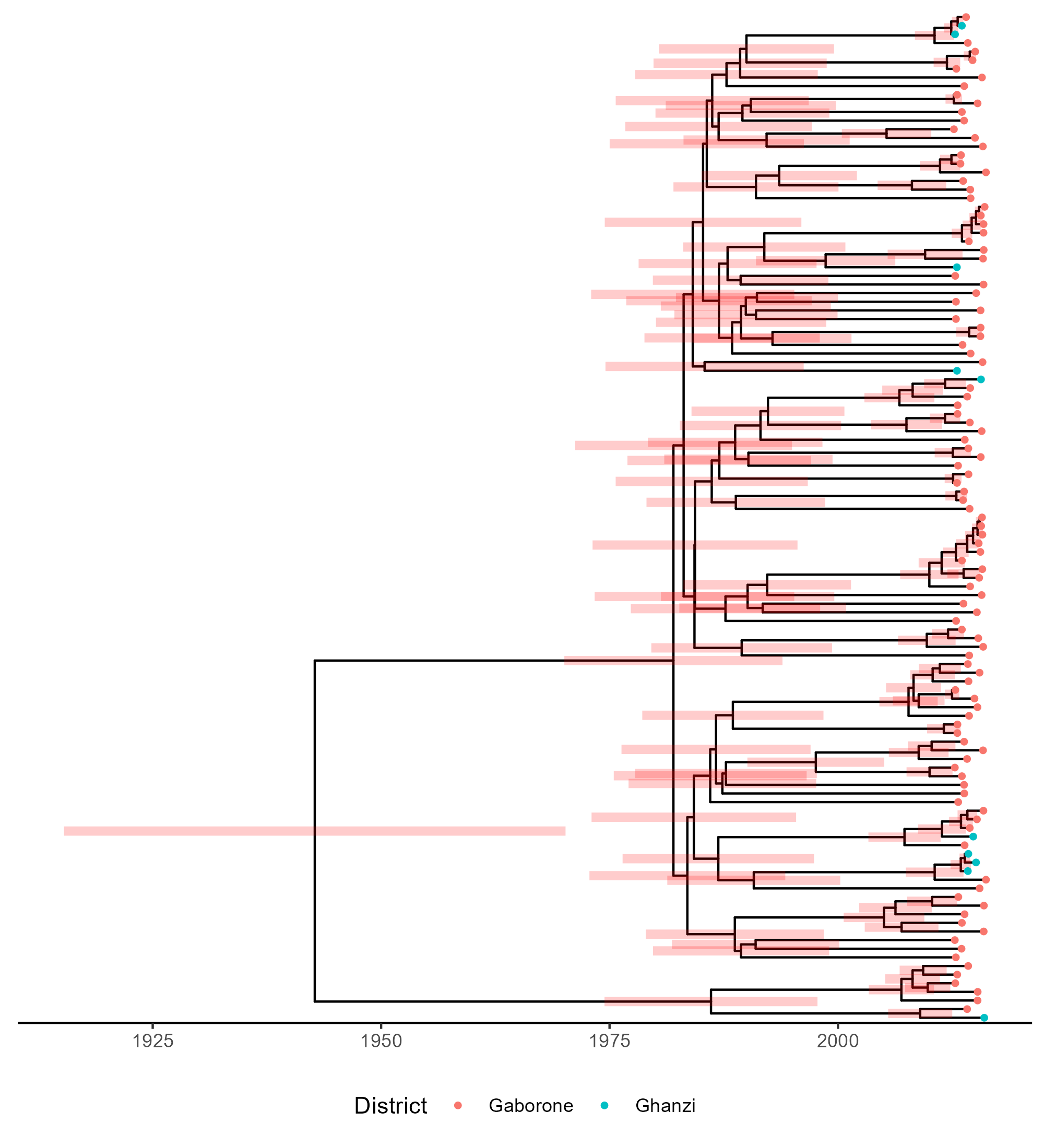


S7 Fig. Maximum clade credibility tree of *Mycobacterium tuberculosis* complex lineage 4.3.4 with 95% highest posterior density intervals of node heights. Tree tips are colored by the location of the sampled isolates.


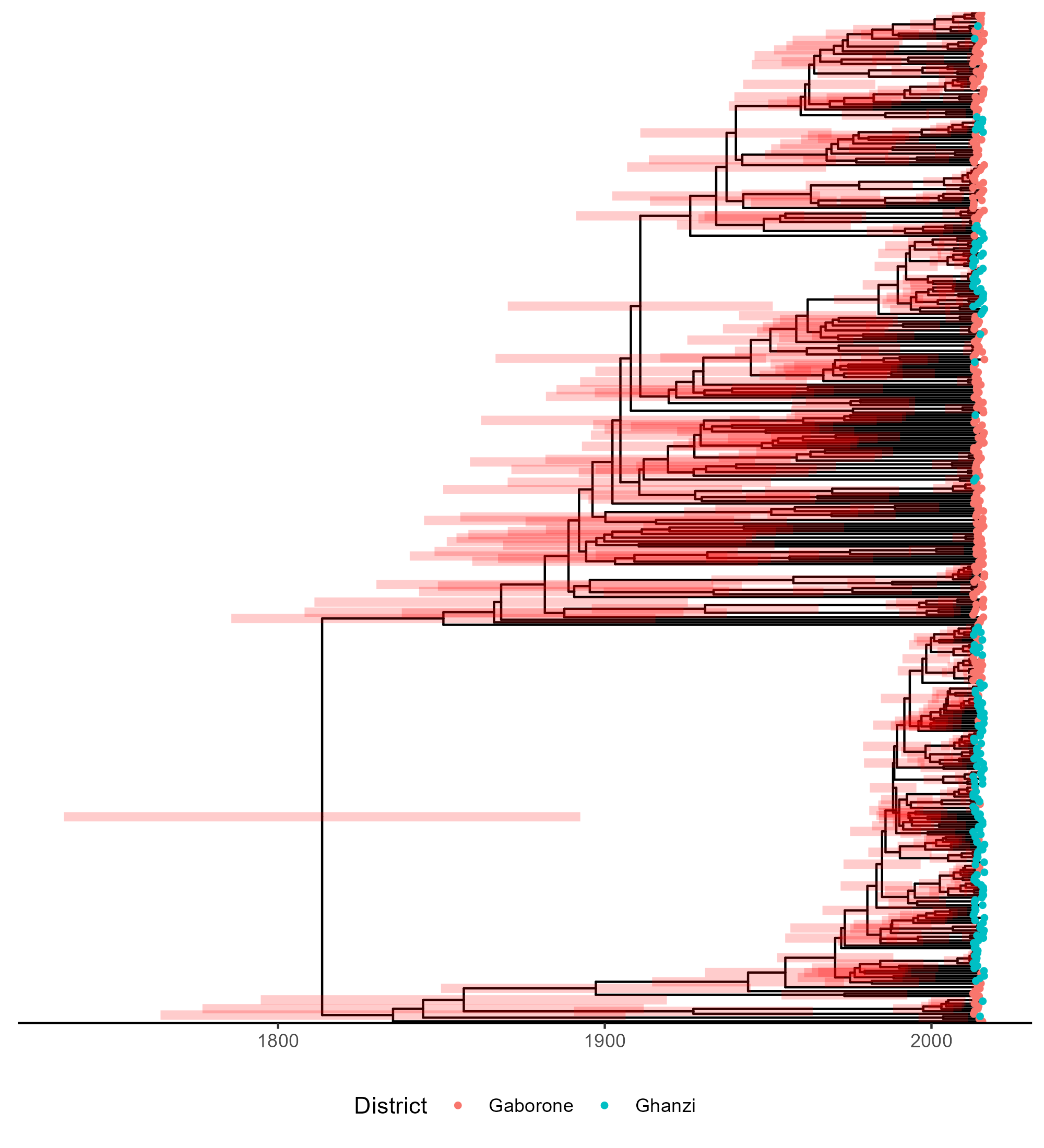


S8 Fig. Maximum clade credibility tree of *Mycobacterium tuberculosis* complex lineage 4.4 with 95% highest posterior density intervals of node heights. Tree tips are colored by the location of the sampled isolates.


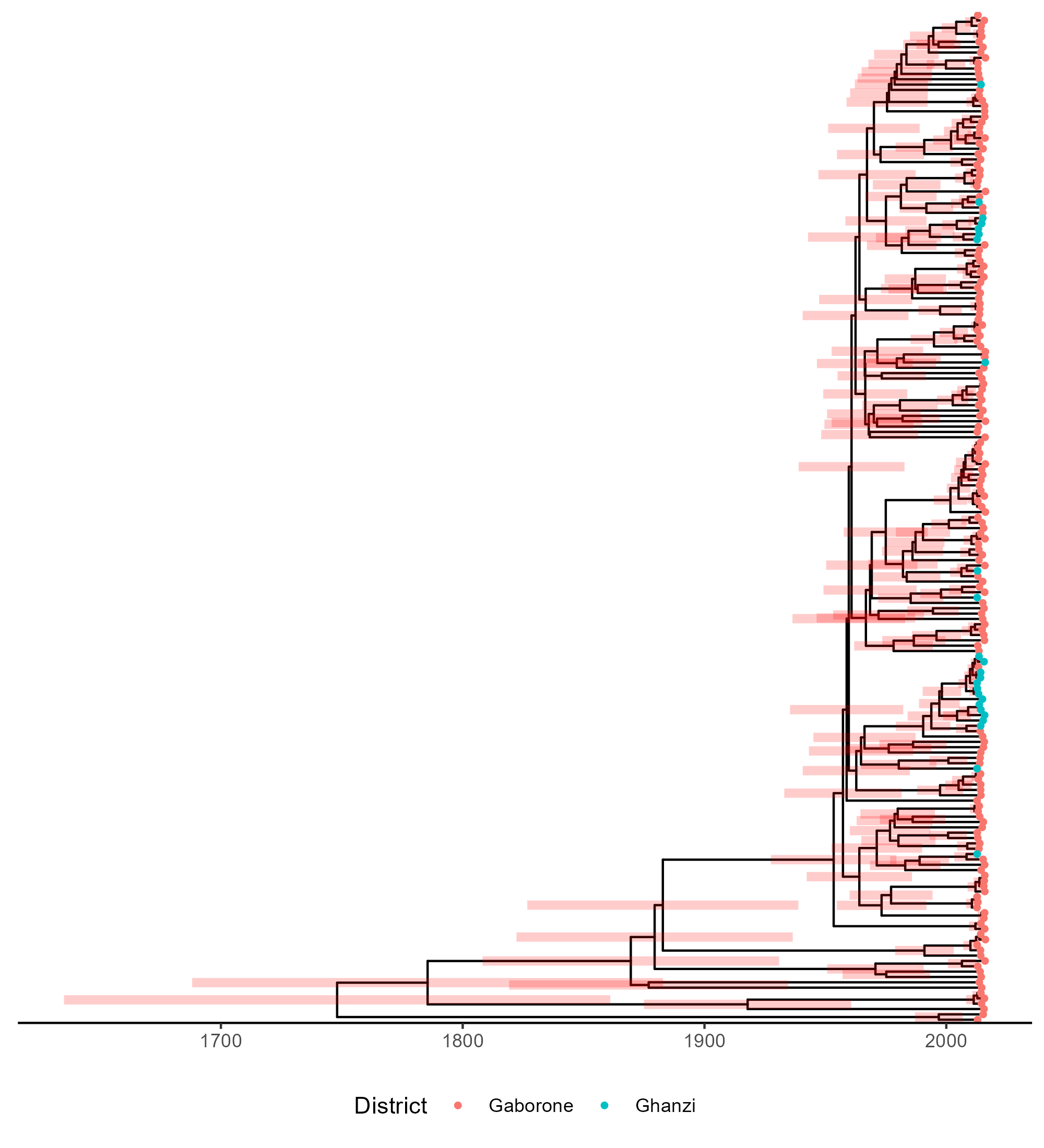


S9 Fig. Maximum clade credibility tree of *Mycobacterium tuberculosis* complex lineage 4.8 with 95% highest posterior density intervals of node heights. Tree tips are colored by the location of the sampled isolates.


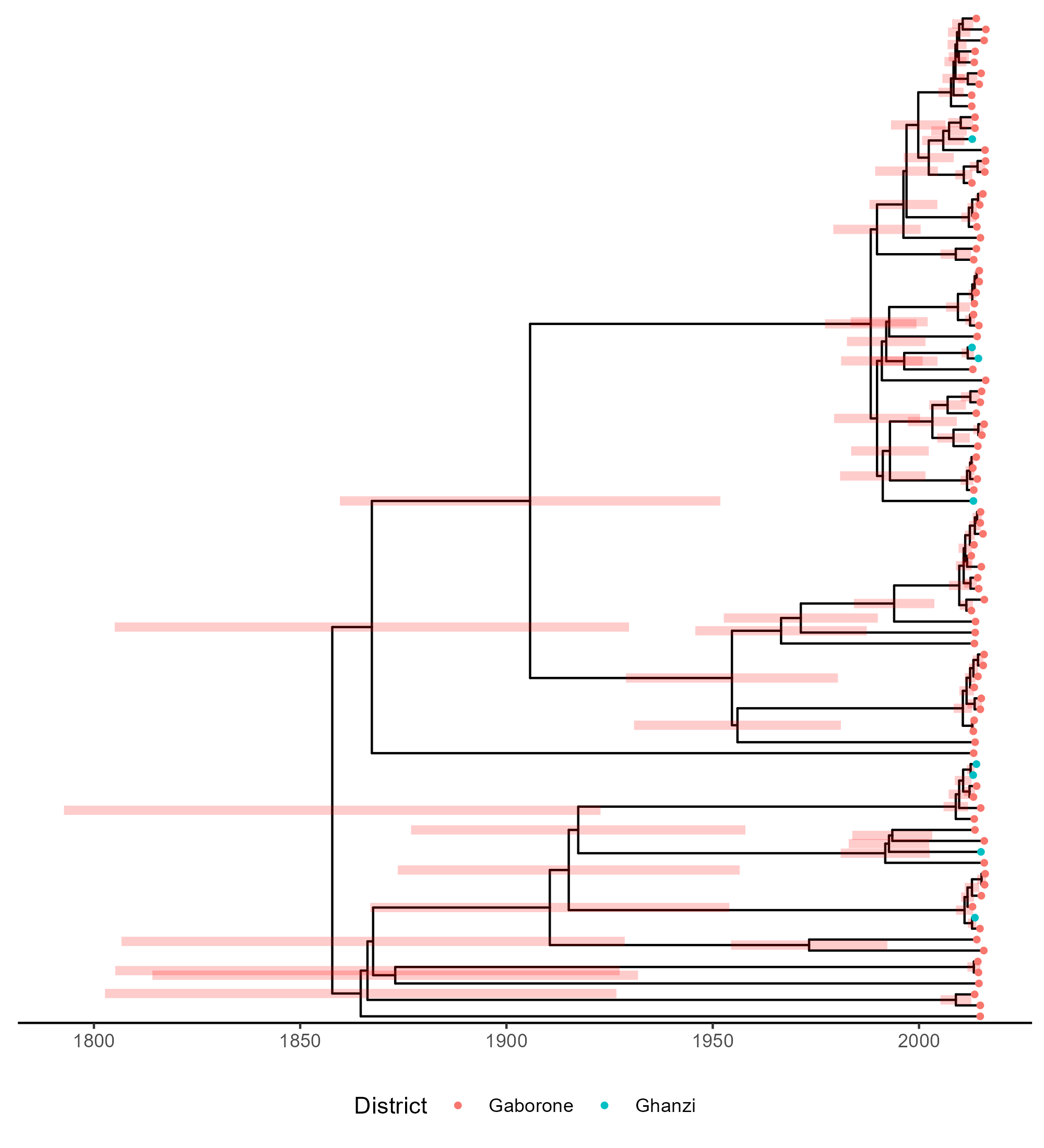


S10 Fig. Maximum likelihood phylogeny of Mycobacterium tuberculosis complex (Mtbc) isolates by sampled location in Botswana, 2012–2016 (n = 1,354).

1. Phylogeny of Lineage 1 Indo-Oceanic Mtbc isolates (n = 86). B. Phylogeny of Lineage 2 East Asian Mtbc isolates (n = 72). C. Phylogeny of Lineage 3 East African-Indian Mtbc isolates (n = 12). D. Phylogeny of Lineage 4 Euro-American Mtbc isolates (n = 1184). Tree tips are colored by the location of the sampled isolates.


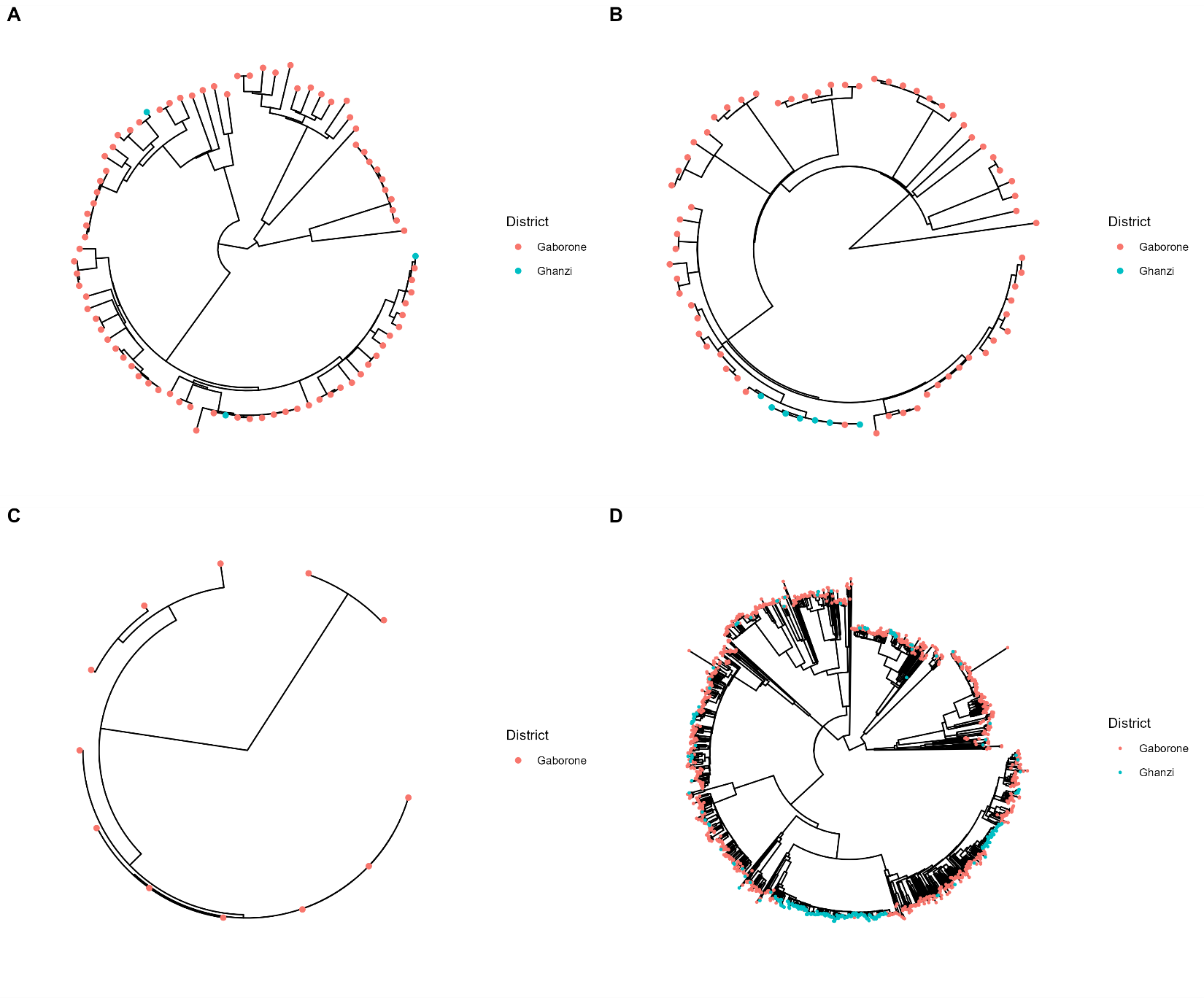


S11 Fig. Cluster size distribution (based on a 5-SNP cutoff) by Mycobacterium tuberculosis complex (Mtbc) lineages and sampling location in Botswana, 2012-2016


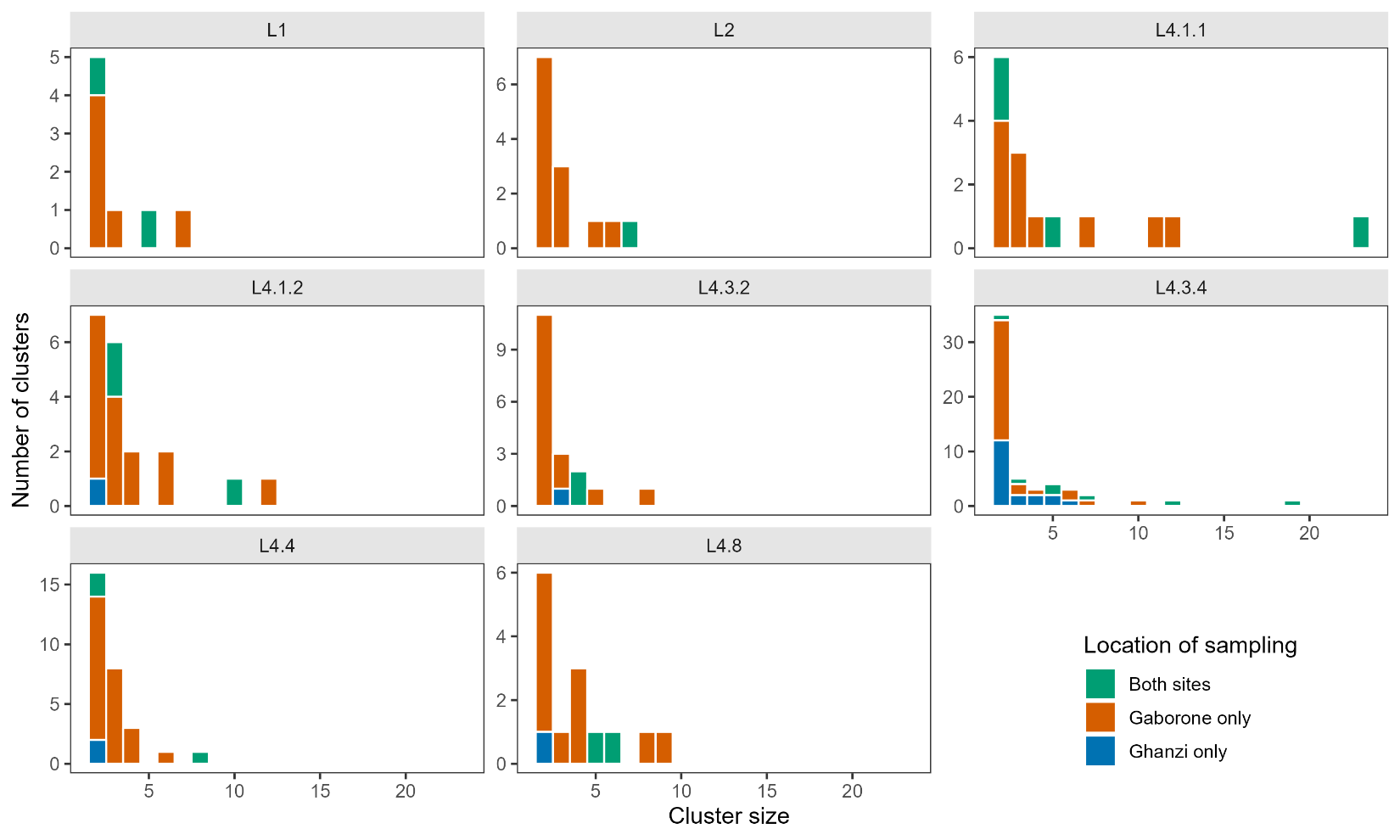


Abbreviations: SNP: single nucleotide polymorphism.

S1 Table. Posterior clock rate of the *Mycobacterium tuberculosis* complex lineages

| **Lineage** | **Clock rate (s/s/y)**  **posterior mean** | **Clock rate (s/s/y)**  **posterior median** | **95% HPD interval** |
| --- | --- | --- | --- |
| 1 | 3.4E-7 | 3.3E-7 | 2.1E-7, 4.6E-7 |
| 2 | 3.7E-7 | 3.6E-7 | 2.4E-7, 5.0E-7 |
| 4.1.1 | 1.4E-7 | 1.4E-7 | 8.0E-8, 2.0E-7 |
| 4.1.2 | 1.3E-7 | 1.3E-7 | 7.8E-8, 1.9E-7 |
| 4.3.2 | 2.0E-7 | 2.0E-7 | 1.3E-7, 2.7E-7 |
| 4.3.4 | 1.0E-7 | 1.0E-7 | 6.5E-8, 1.4E-7 |
| 4.4 | 1.5E-7 | 1.5E-7 | 9.2E-8, 2.1E-7 |
| 4.8 | 2.0E-7 | 2.0E-7 | 1.3E-7, 2.9E-7 |

Abbreviations: s/s/y: substitutions per site per year. HPD: highest posterior density.

S2 Table. Genomic cluster proportions (based on a 12-SNPs cutoff) of the *Mycobacterium tuberculosis* complex lineages

| **Lineage** | **Cluster proportions** | **Crude OR (95% CI)** | **Adjusted^1^ OR (95% CI)** |
| --- | --- | --- | --- |
| L1 | 38/86 | 1 | 1 |
| L2 | 57/72 | 4.80 (2.40, 10.01) | 4.44 (2.19, 9.37) |
| L4.1.1 | 103/141 | 3.42 (1.96, 6.07) | 3.48 (1.97, 6.25) |
| L4.1.2 | 96/149 | 2.29 (1.34, 3.95) | 2.08 (1.20, 3.64) |
| L4.3.2 | 79/117 | 2.63 (1.48, 4.70) | 2.52 (1.41, 4.55) |
| L4.3.4 | 260/400 | 2.35 (1.47, 3.78) | 1.49 (0.91, 2.47) |
| L4.4 | 124/189 | 2.41 (1.44, 4.08) | 2.21 (1.30, 3.79) |
| L4.8 | 73/92 | 4.85 (2.54, 9.57) | 4.32 (2.24, 8.62) |

Abbreviations: SNP: single nucleotide polymorphism. OR: odds ratio. CI: confidence interval.

^1^Adjusted for age, gender, HIV status, and district.

S3 Table. HIV prevalence by *Mycobacterium tuberculosis* complex lineages in Botswana, 2012 – 2016.

| **Lineage** | **HIV prevalence^1^** | **Crude OR (95% CI)** | **Adjusted^2^ OR (95% CI)** |
| --- | --- | --- | --- |
| L1 | 56/83 | 1 | 1 |
| L2 | 42/69 | 0.75 (0.38, 1.46) | 0.69 (0.33, 1.44) |
| L4.1.1 | 71/140 | 0.50 (0.28, 0.87) | 0.53 (0.28, 0.99) |
| L4.1.2 | 95/147 | 0.88 (0.49, 1.55) | 1.01 (0.54, 1.90) |
| L4.3.2 | 72/115 | 0.81 (0.44, 1.46) | 0.89 (0.46, 1.71) |
| L4.3.4 | 182/381 | 0.44 (0.26, 0.72) | 0.67 (0.38, 1.17) |
| L4.4 | 104/184 | 0.63 (0.36, 1.07) | 0.64 (0.35, 1.16) |
| L4.8 | 42/89 | 0.43 (0.23, 0.80) | 0.45 (0.22, 0.88) |

Abbreviations: OR: odds ratio. CI: confidence interval.

^1^Exclude participants with unknown HIV status (n = 38).

^2^Adjusted for age, gender, and district.

S4 Table. Unique single nucleotide polymorphisms (SNPs) distinguishing *Mycobacterium tuberculosis* complex lineages L4.3.2 and L4.3.4 in Botswana.

| **L4.3.2** | | |
| --- | --- | --- |
| **Gene** | **Position** | **Mutation** |
| Rv0018c | 21795 | P463S (ccg/Tcg) |
| Rv0043c | 47699 | R134R (cgc/cgT) |
| Rv0092 | 102188 | R536S (cgc/Agc) |
| Rv0094c | 103836 | N276K (aac/aaA) |
| Rv0094c | 103879 | G262D (ggc/gAc) |
| Rv0101 | 113838 | L1280F (ctt/Ttt) |
| Rv0134 | 162581 | G271S (ggc/Agc) |
| Rv0204c | 243118 | R33C (cgc/Tgc) |
| Rv0205 | 243535 | A51V (gcg/gTg) |
| Rv0206c | 244911 | P803R (cca/cGa) |
| Rv0237 | 287904 | V240A (gtc/gCc) |
| Rv0270 | 326039 | A491A (gcc/gcT) |
| Rv0376c | 453780 | M198T (atg/aCg) |
| Rv0386 | 466096 | R896C (cgc/Tgc) |
| Rv0425c | 515125 | N66T (aac/aCc) |
| Rv0444c | 533781 | E3E (gaa/gaG) |
| Rv0493c | 584438 | P85S (ccc/Tcc) |
| Rv0554 | 646243 | S259S (agt/agC) |
| Rv0610c | 705595 | A105A (gcg/gcA) |
| Rv0721 | 814641 | V105A (gta/gCa) |
| Rv0750 | 842111 | L27V (ctg/Gtg) |
| Rv0758 | 853469 | G358G (ggc/ggA) |
| Rv0881 | 980100 | R247W (cgg/Tgg) |
| Rv0882 | 980360 | G46C (ggt/Tgt) |
| Rv0938 | 1047683 | L516F (ttg/ttT) |
| Rv0976c | 1090172 | R2C (cgt/Tgt) |
| Rv1028c | 1149551 | E712E (gag/gaA) |
| Rv1161 | 1290326 | R1000H (cgt/cAt) |
| Rv1186c | 1328687 | P207A (ccc/Gcc) |
| Rv1249c | 1393626 | L119L (ctt/ctC) |
| Rv1286 | 1440150 | L415S (tta/tCa) |
| Rv1417 | 1592453 | S102P (tcg/Ccg) |
| Rv1417 | 1592454 | S102_ (tcg/tAg) |
| Rv1446c | 1625019 | P116L (ccc/cTc) |
| Rv1501 | 1692141 | I84I (ata/atC) |
| Rv1502 | 1693561 | Y213C (tac/tGc) |
| Rv1508c | 1698911 | G328G (ggc/ggT) |
| Rv1514c | 1706119 | S159S (tca/tcG) |
| Rv1518 | 1710070 | Q143E (caa/Gaa) |
| Rv1524 | 1718761 | G12G (ggc/ggT) |
| Rv1571 | 1778557 | L7L (ctg/Ttg) |
| Rv1587c | 1788570 | N198K (aac/aaA) |
| Rv1587c | 1788613 | G184D (ggc/gAc) |
| Rv1599 | 1799806 | A75V (gcg/gTg) |
| Rv1614 | 1813255 | C29S (tgc/Agc) |
| Rv1633 | 1838153 | R360H (cgc/cAc) |
| Rv1662 | 1885073 | M1124V (atg/Gtg) |
| Rv1811 | 2053682 | I80I (atc/atT) |
| Rv1870c | 2121063 | G123D (ggt/gAt) |
| Rv1938 | 2191251 | K75K (aag/aaA) |
| Rv1939 | 2192350 | A86V (gcc/gTc) |
| Rv1945 | 2196715 | V243L (gtc/Ctc) |
| Rv1945 | 2196964 | N326H (aac/Cac) |
| Rv1945 | 2197065 | A359A (gcc/gcG) |
| Rv1945 | 2197278 | H430H (cat/caC) |
| Rv2003c | 2249403 | R6R (cgg/cgA) |
| Rv2176 | 2438090 | M50I (atg/atA) |
| Rv2264c | 2537602 | L250R (ctt/cGt) |
| Rv2273 | 2546359 | R86R (cgc/cgT) |
| Rv2368c | 2649855 | D40D (gac/gaT) |
| Rv2379c | 2659711 | P792L (ccg/cTg) |
| Rv2398c | 2695094 | A236P (gcc/Ccc) |
| Rv2582 | 2907629 | Q272Q (cag/caA) |
| Rv2605c | 2932318 | G275G (ggt/ggG) |
| Rv2637 | 2964024 | V147I (gtc/Atc) |
| Rv2690c | 3007840 | V457A (gtg/gCg) |
| Rv2737c | 3051068 | Q119Q (cag/caA) |
| Rv2799 | 3107859 | A31V (gcg/gTg) |
| Rv2823c | 3130682 | G364G (ggc/ggT) |
| Rv2886c | 3195557 | H292H (cac/caT) |
| Rv2886c | 3195975 | A153V (gcg/gTg) |
| Rv2931 | 3247874 | R810R (cgg/cgA) |
| Rv2931 | 3247877 | F811F (ttt/ttC) |
| Rv2941 | 3284726 | L464L (ctc/ctG) |
| Rv2984 | 3340615 | F254L (ttc/ttG) |
| Rv2998 | 3357160 | A116P (gcg/Ccg) |
| Rv3042c | 3401951 | V404V (gtc/gtG) |
| Rv3053c | 3414791 | A56A (gcc/gcG) |
| Rv3072c | 3436296 | D9D (gat/gaC) |
| Rv3085 | 3451670 | V251L (gtg/Ctg) |
| Rv3092c | 3460986 | P250L (ccg/cTg) |
| Rv3093c | 3462135 | C210W (tgc/tgG) |
| Rv3121 | 3486977 | K157E (aaa/Gaa) |
| Rv3130c | 3495658 | P237A (cct/Gct) |
| Rv3151 | 3518167 | I474M (ata/atG) |
| Rv3195 | 3564790 | D143N (gac/Aac) |
| Rv3201c | 3576965 | P24P (ccc/ccT) |
| Rv3224A | 3600717 | I28S (atc/aGc) |
| Rv3302c | 3688944 | G167S (ggc/Agc) |
| Rv3335c | 3722592 | E3D (gag/gaC) |
| Rv3359 | 3771563 | G74C (ggc/Tgc) |
| Rv3375 | 3788840 | A74S (gcc/Tcc) |
| Rv3467 | 3884748 | G262D (ggc/gAc) |
| Rv3467 | 3884791 | N276K (aac/aaA) |
| Rv3591c | 4033954 | S30F (tcc/tTc) |
| Rv3610c | 4050877 | A669A (gcc/gcG) |
| Rv3685c | 4127997 | R243R (cgc/cgG) |
| Rv3698 | 4141036 | D182Y (gat/Tat) |
| Rv3705c | 4148529 | D145A (gac/gCc) |
| Rv3764c | 4210274 | C246R (tgt/Cgt) |
| Rv3776 | 4222073 | M329V (atg/Gtg) |
| Rv3779 | 4225647 | A221A (gcg/gcA) |
| Rv3797 | 4251087 | M1I (atg/atA) |
| Rv3824c | 4293072 | L35F (ctt/Ttt) |
| Rv3825c | 4295631 | V1325V (gtg/gtT) |
| Rv3825c | 4297793 | T605A (acc/Gcc) |
| Rv3831 | 4306155 | S133S (agc/agT) |
| Rv3871 | 4349688 | P288S (ccc/Tcc) |
| Rv3871 | 4350446 | S540S (tcg/tcC) |
| Rv3903c | 4389202 | D411N (gac/Aac) |
| **L4.3.4** | | |
| **Gene** | **Position** | **Mutation** |
| Rv0015c | 17608 | S385R (agc/agG) |
| Rv0092 | 101727 | G382E (gga/gAa) |
| Rv0095c | 104962 | A85V (gcg/gTg) |
| Rv0095c | 105045 | D57E (gac/gaG) |
| Rv0149 | 176303 | H202Y (cac/Tac) |
| Rv0197 | 234051 | P607P (ccg/ccA) |
| Rv0236c | 285871 | V327V (gtt/gtC) |
| Rv0399c | 478358 | E67K (gag/Aag) |
| Rv0466 | 557133 | V226I (gtt/Att) |
| Rv0483 | 571943 | V78V (gtg/gtA) |
| Rv0515 | 608037 | H496P (cac/cCc) |
| Rv0574c | 667659 | D246N (gac/Aac) |
| Rv0638 | 733798 | S21T (agc/aCc) |
| Rv0642c | 736710 | N165S (aac/aGc) |
| Rv0690c | 790180 | D298D (gat/gaC) |
| Rv0815c | 909280 | A13A (gct/gcG) |
| Rv0930 | 1037355 | T119T (act/acC) |
| Rv0933 | 1041445 | T61M (acg/aTg) |
| Rv0948c | 1057788 | K59T (aag/aCg) |
| Rv0959 | 1071797 | G181G (ggc/ggG) |
| Rv0969 | 1079927 | T395T (acc/acA) |
| Rv0970 | 1081681 | V210V (gtt/gtC) |
| Rv1155 | 1281443 | V5V (gtc/gtG) |
| Rv1166 | 1297327 | V392V (gtg/gtA) |
| Rv1166 | 1297999 | S616S (tct/tcG) |
| Rv1278 | 1428506 | A365S (gcc/Tcc) |
| Rv1288 | 1441533 | D62E (gac/gaG) |
| Rv1411c | 1588456 | R9R (cgt/cgC) |
| Rv1536 | 1736638 | R40R (cgc/cgT) |
| Rv1569 | 1777213 | A171G (gcc/gGc) |
| Rv1639c | 1847811 | P216P (cct/ccG) |
| Rv1872c | 2123145 | V3I (gtc/Atc) |
| Rv1877 | 2126366 | V155L (gtc/Ctc) |
| Rv1884c | 2134215 | H16R (cac/cGc) |
| Rv1969 | 2213265 | R137R (cgg/cgA) |
| Rv1987 | 2231132 | S36N (agt/aAt) |
| Rv1992c | 2237053 | P85L (cct/cTt) |
| Rv2008c | 2257780 | I55L (atc/Ctc) |
| Rv2017 | 2264782 | A262E (gcg/gAg) |
| Rv2024c | 2269780 | D154G (gac/gGc) |
| Rv2024c | 2270102 | W47R (tgg/Cgg) |
| Rv2252 | 2527676 | G230S (ggc/Agc) |
| Rv2326c | 2599821 | A43S (gcg/Tcg) |
| Rv2380c | 2664299 | T939T (acc/acG) |
| Rv2402 | 2698585 | Y19Y (tac/taT) |
| Rv2419c | 2717710 | L30L (ttg/ttA) |
| Rv2531c | 2857014 | T256T (acc/acT) |
| Rv2547 | 2868659 | A18A (gcc/gcG) |
| Rv2570 | 2894854 | Q115_ (cag/Tag) |
| Rv2582 | 2906918 | L35L (cta/ctT) |
| Rv2617c | 2946157 | N44S (aat/aGt) |
| Rv2716 | 3029610 | A147T (gcg/Acg) |
| Rv2790c | 3099269 | F301V (ttc/Gtc) |
| Rv2897c | 3207297 | L216M (ctg/Atg) |
| Rv2905 | 3214790 | L55L (ctg/Ttg) |
| Rv2959c | 3312632 | W69_ (tgg/tgA) |
| Rv3057c | 3418328 | D112A (gat/gCt) |
| Rv3057c | 3418330 | H111H (cac/caT) |
| Rv3117 | 3484012 | A13A (gct/gcG) |
| Rv3136A | 3503231 | A16G (gcg/gGg) |
| Rv3236c | 3612009 | A370T (gcc/Acc) |
| Rv3463 | 3881187 | G94D (ggc/gAc) |
| Rv3598c | 4042761 | D60D (gac/gaT) |
| Rv3667 | 4109354 | L521L (cta/ctC) |
| Rv3703c | 4146330 | L188L (ttg/Ctg) |
| Rv3838c | 4311871 | H267Y (cac/Tac) |
| Rv3888c | 4372661 | I16V (atc/Gtc) |
| Rv3897c | 4383094 | C183R (tgt/Cgt) |
| Rv3909 | 4395964 | T591T (acc/acA) |
